# Supplementary figures and images for: A Microfluidic Platform for High-Throughput Multiplexed Protein Quantitation
Source: PLoS One. 2015 Feb 13;10(2):e0117744. doi: 10.1371/journal.pone.0117744 (PMC4334502; doi:10.1371/journal.pone.0117744)

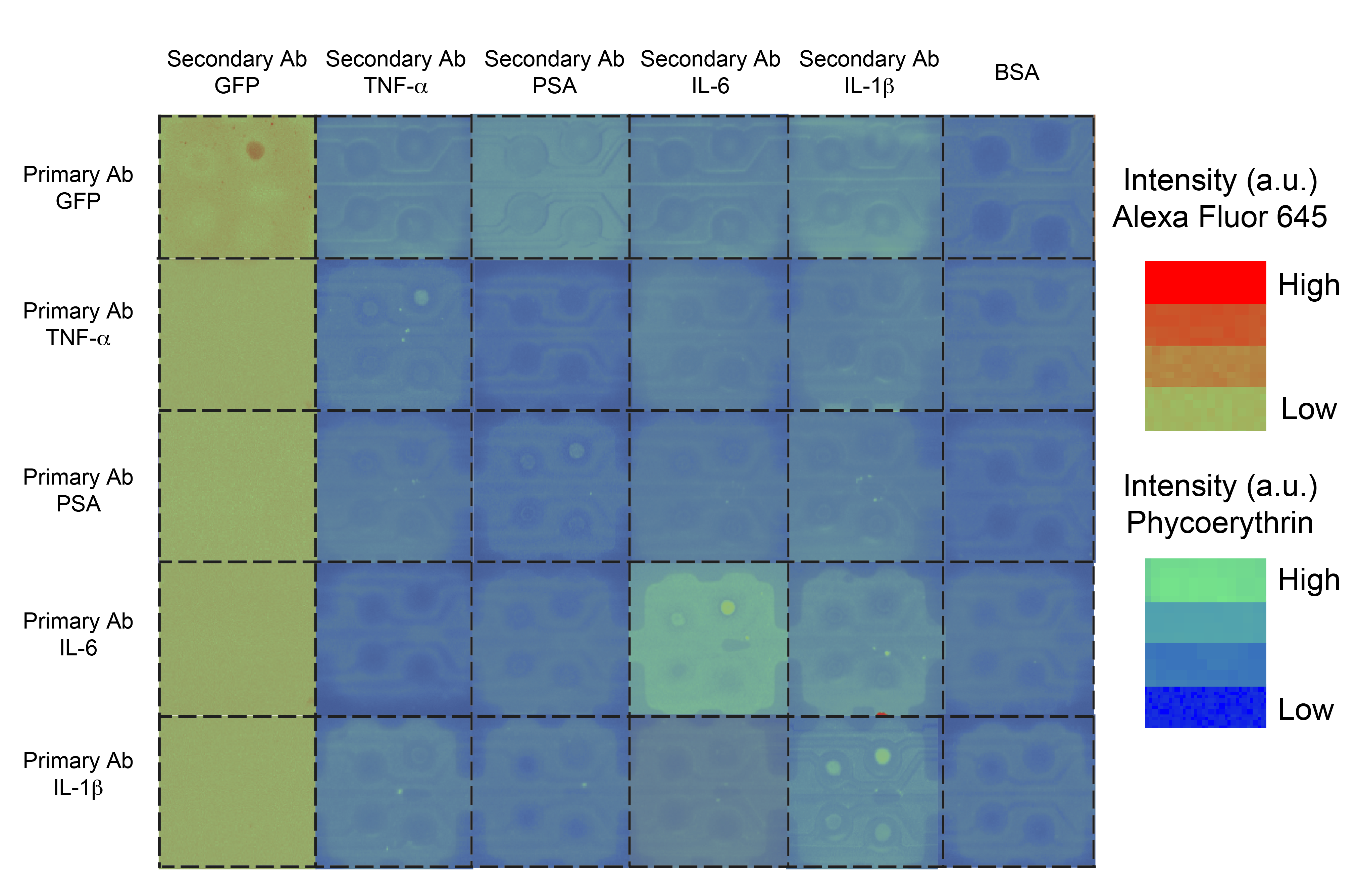

Supplement: S1 Fig — All combinations of the five different primary antibodies and secondary antibodies were tested. A negative control (2% BSA in PBS) and a cocktail of proteins in buffer (GFP, IL-6, IL-1β, TNF-α, and PSA), at three different concentrations (5, 20, 100pM), were sequentially flowed. The color bars indicate the relative fluorescence intensity. (TIF) [file pone.0117744.s001.tif]

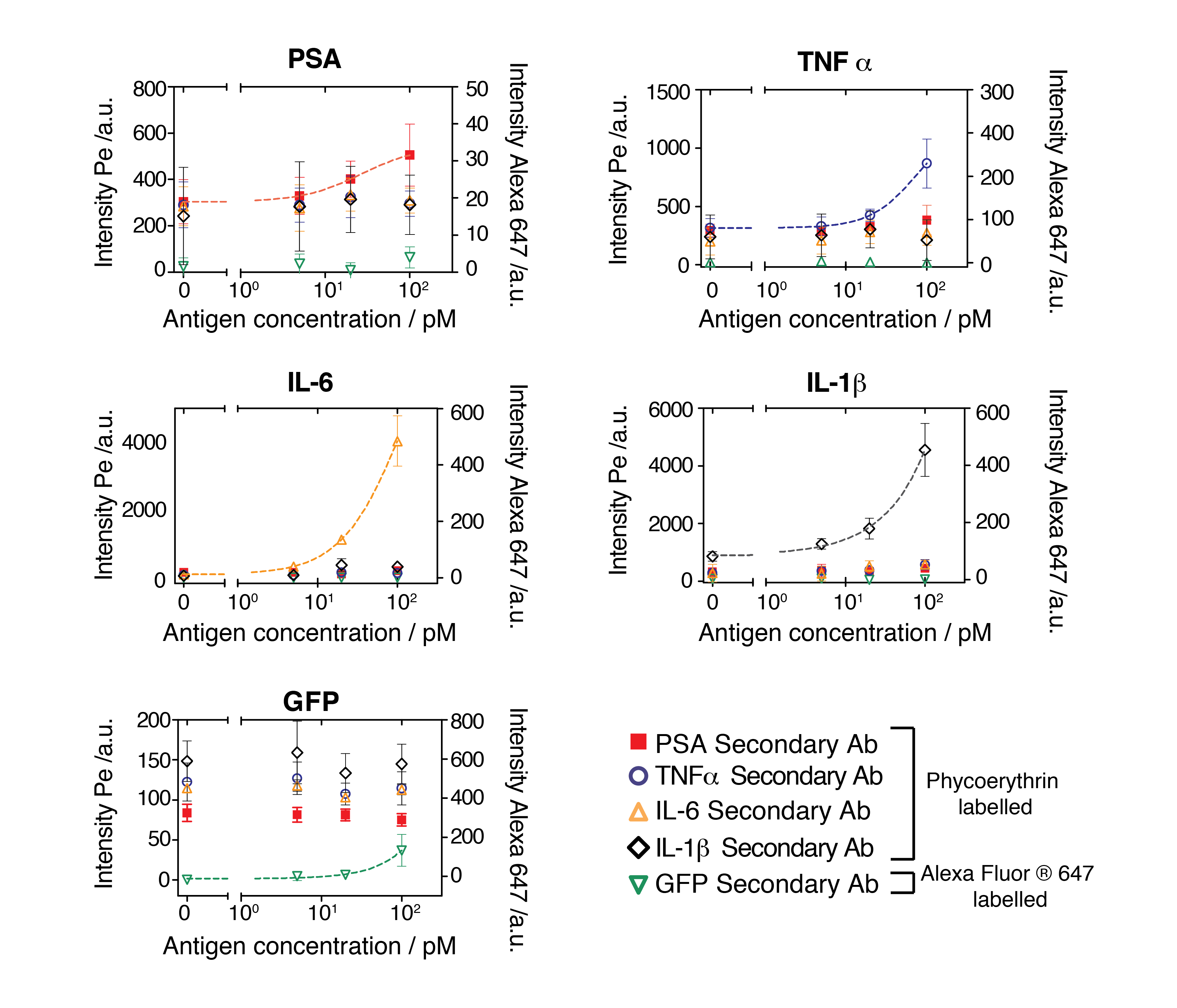

Supplement: S2 Fig — For each graph the primary antibody was specific only for one antigen and the different curves are related to specific and non-specific secondary antibodies for the antigen. The secondary antibodies were spotted at a concentration of 2 nM. Left y-axis refers to secondary antibody labeled with phycoerythrin (Pe) and the right y-axis to secondary antibody labeled with Alexa Fluor 647 (error bars are std. dev., n = 4–7). (TIF) [file pone.0117744.s002.tif]

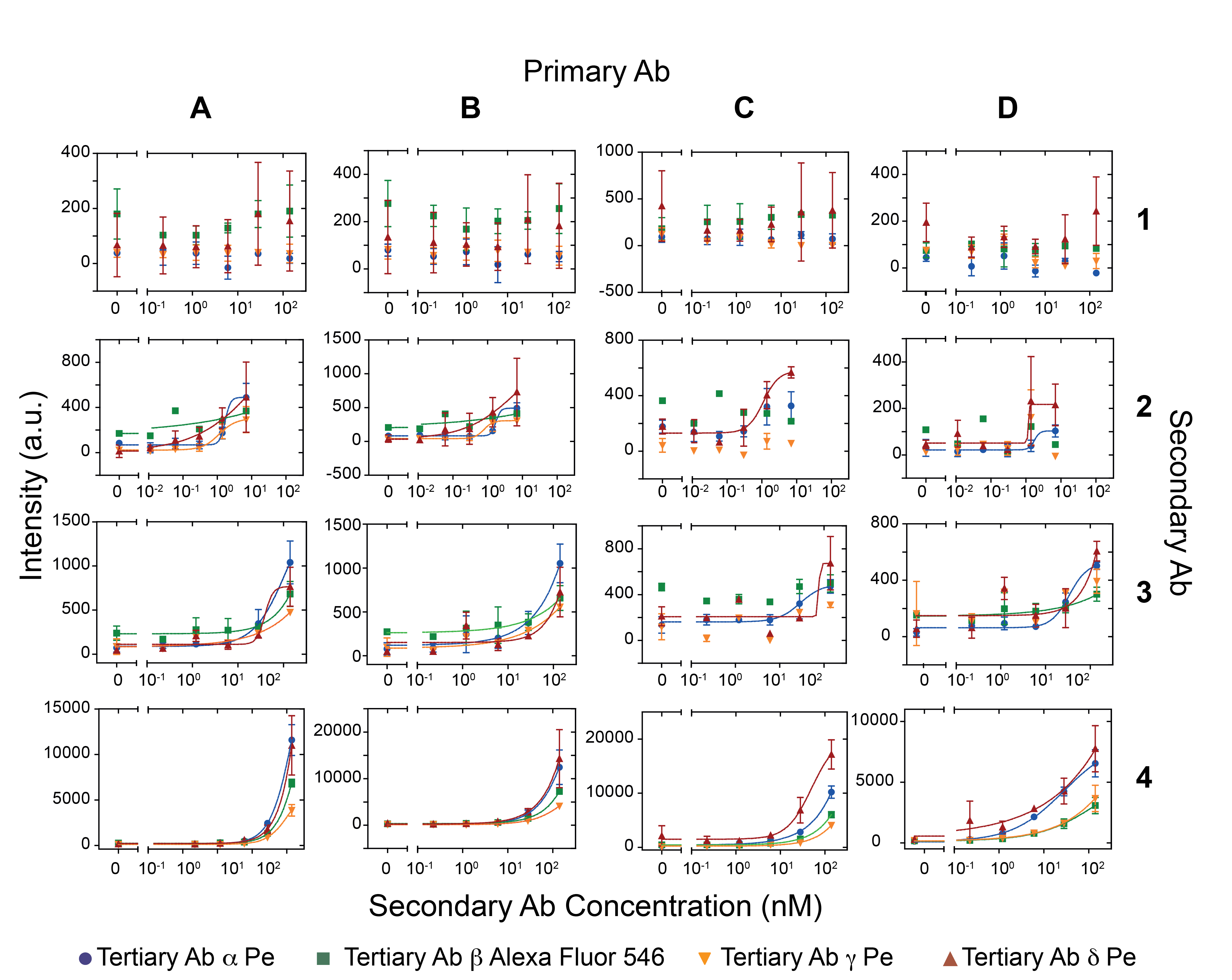

Supplement: S3 Fig — Different combinations of four secondary antibodies (1–4) and four tertiary antibodies (α-δ) were spotted. The secondary antibodies were spotted at six different concentrations, and the tertiary antibody at a concentration of 30 nM. After surface derivatization, four different primary antibodies (A-D) were flowed in sequence, one for each button, followed by 100 pM PSA in a buffer solution. Next the secondary antibody was allowed to diffuse in the reaction chamber and finally, the tertiary (α-δ) antibodies diffused in reaction chamber. (TIF) [file pone.0117744.s003.tif]

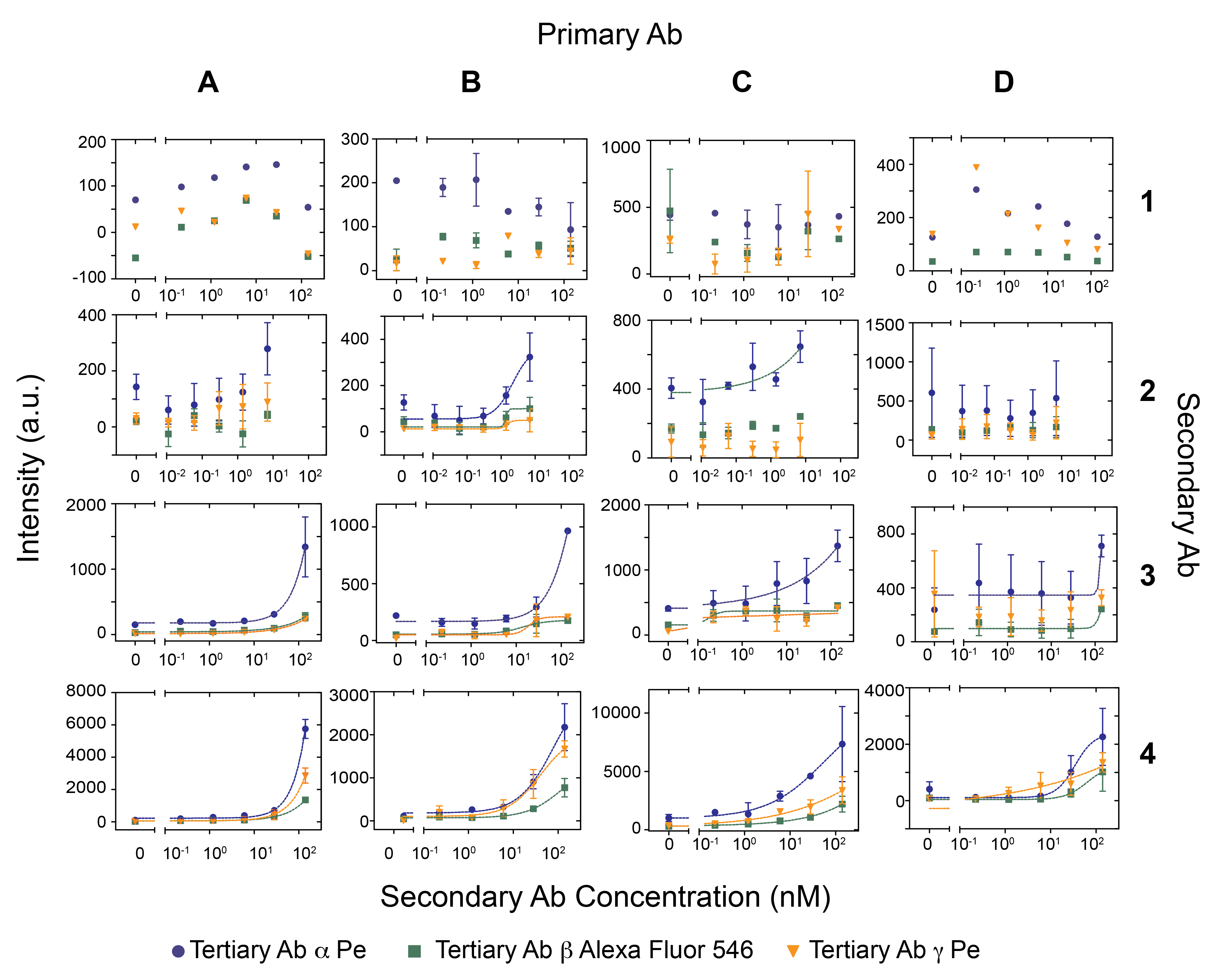

Supplement: S4 Fig — 16 different combinations of antibodies were spotted: four primary antibodies (A-B) and four secondary antibodies (1–4). The secondary antibodies were spotted at six different concentrations (0, 0.2, 1.2, 6, 28 and 140 nM), and the primary antibody at a concentration of 600 nM. After the surface derivatization, the primary antibody was allowed to diffuse and immobilize to the surface, then 100 pM PSA was flowed in the chip. Next the secondary antibody was allowed to diffuse in the reaction chamber and finally, three different tertiary antibodies (α, β, γ) were sequentially flowed, one for each button. (TIF) [file pone.0117744.s004.tif]

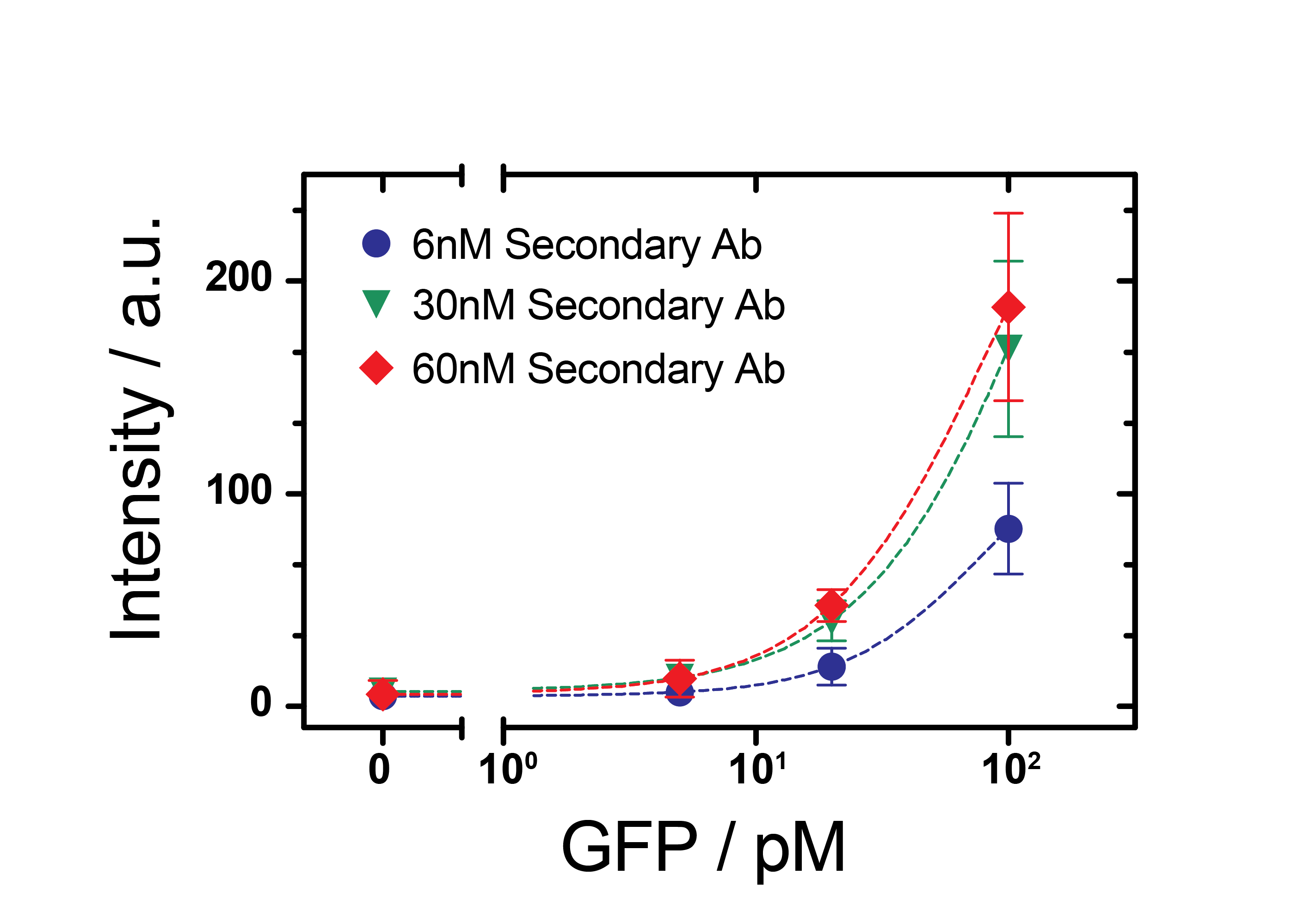

Supplement: S5 Fig — The control lines were filled with oil (Fluorinert FC-40), and the functionalization of the surface was performed, as described. Next, the flow layer was dried by pushing air through the channel at 3.5 psi for 30 min. The chip was then stored in a vacuum chamber at room temperature for 1 day. To perform the immunoassay the chambers containing the spotted antibodies against GFP were filled with PBS in order to re-hydrate the spots. The unit cells were then isolated and the primary antibody allowed to diffuse into the reaction chamber. After a washing step, four different concentrations of GFP were flowed in sequence, one for each button. Finally, the secondary antibody was allowed to diffuse into the reaction chamber and the device was scanned. (TIF) [file pone.0117744.s005.tif]

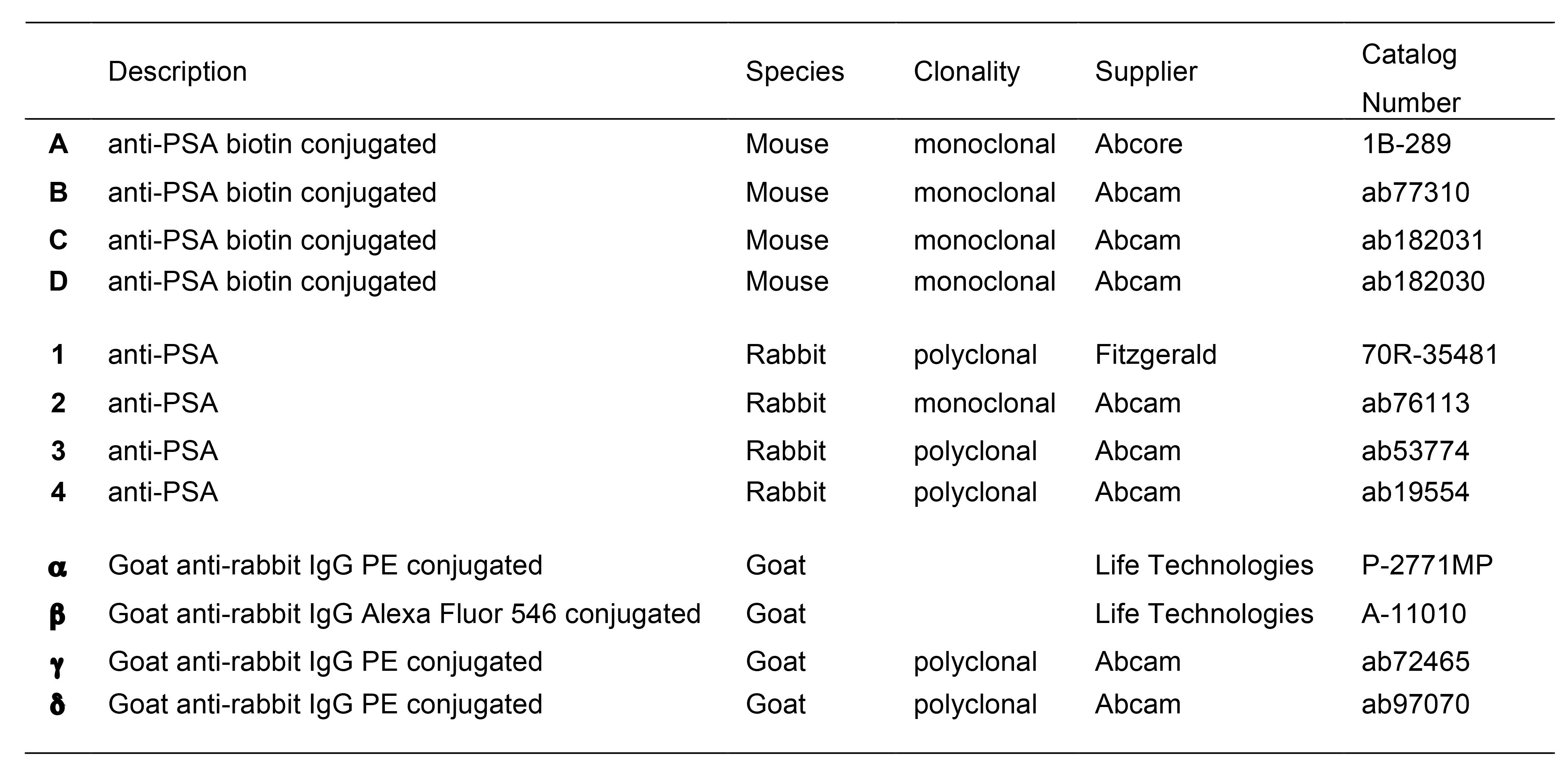

Supplement: S1 Table — The table lists the 12 antibodies tested. Description, species, clonality, supplier and catalog number are provided for each antibody (Life-Technologies did not provide the clonality for antibodies α and β but they are likely polyclonal antibodies). The first set (A-D) includes the biotinylated primary antibodies, the second set (1–4) the secondary antibodies, and the third set (α-δ) the tertiary antibodies conjugated with phycoerythrin or Alexa Fluor 546. (TIF) [file pone.0117744.s006.tif]
